# Possibility of estimating future mutants for influenza: Comparison between previous prediction and subsequent years observation

**DOI:** 10.3389/fmicb.2022.1031672

**Published:** 2022-10-05

**Authors:** Tiantian Mao, Deyu Yan, Mengdi Zhou, Tianyi Qiu, Zhiwei Cao

**Affiliations:** ^1^Department of Gastroenterology, Shanghai Tenth People’s Hospital, School of Life Sciences and Technology, Tongji University, Shanghai, China; ^2^Institute of Clinical Science, Zhongshan Hospital, Shanghai Medical College, Fudan University, Shanghai, China; ^3^School of Life Sciences, Fudan University, Shanghai, China

**Keywords:** influenza, hemagglutinin (HA) protein, antigenic region, mutation, mutation profile

## Introduction

Despite less cases being reported in recent years, the seasonal influenza remains a concern because of constant genomic mutation and potential antigenic evasion, particularly in surface hemagglutinin (HA) (Petrova and Russell, 2018). Though World Health Organization makes periodic surveillance on circulating strains and recommends the next vaccine components when new variants are detected, months are often needed for vaccine manufacturing, packaging, and distribution (Sabbaghi et al., 2019). This may sometimes cause mismatch between vaccinated components and circulating ones hitting months later, leading to occasional vaccine failure or reduced effectiveness (Poland, 2018). While the long-term and broadly protection vaccines are explored, predicting the future mutational spectrum may be helpful for better preparation and protection.

In recent years, some interesting work tried in this direction with encouraging results obtained (Salama et al., 2016; Yin et al., 2020). In 2016, we proposed a “mutation-selection-ranking” strategy to explore the possibility to pre-estimate the single-site mutational profiles in influenza HA antigenic sites and gave a prediction list for A/H1N1 (Xu et al., 2016). The model was built on 5 well-established antigenic regions (AR) via data between 1918 and 1998. Independent validation on data between 1999 and 2014 showed that over 94% of the newly emerged antigenic sites were covered by predicted profile. At last, based on the template strain of A/H1N1 (A/Alberta/47/2014), a prediction list of future variants was also published at the end of the article, which now has become an ideal test to re-evaluate the prediction possibility. Here, we collected the observed HA mutants before the SARS-CoV-2 outbreak from 2015 to 2019 for annual comparison with the predicted list.

## High agreement between prediction and observation by retrospective comparison

According to the Supplementary Table 4 in previous article (Xu et al., 2016), candidate sequences were predicted for 5 ARs, with 20 Ca1, 20 Ca2, 10 Cb, 50 Sa, and 50 Sb variants, respectively. In terms of validation, the three parameters were again taken as previously introduced, covering the type coverage, strain coverage, and the ranking list of mutants in each AR. Basically, the high strain coverage, low type coverage, and top-ranking indicate the successful capture of the future dominant mutants from a large number of observed variants (see Supplementary Methods).

**FIGURE 1:**
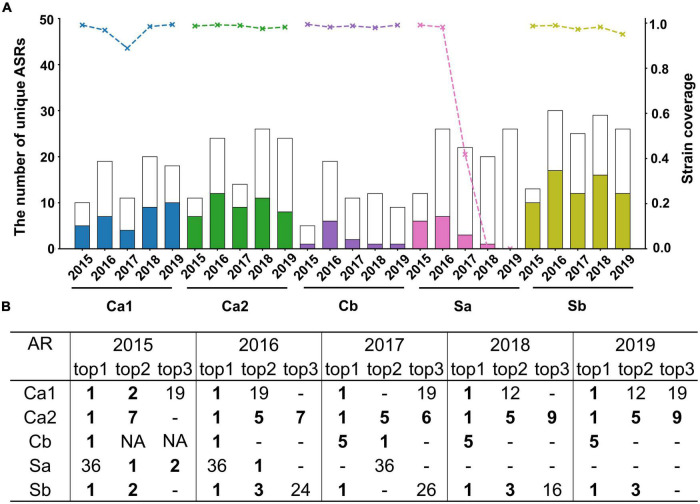
Prediction performance of A/H1N1 during 2015–2019. **(A)** Type coverage and strain coverage of five ARs. Each bar shows the total number of unique AR sequences observed in the circulation year, with the colored portion indicating the number of successfully predicted ARs, corresponding to the left axis. The top line indicates the strain coverage for each year, corresponding to the right axis. **(B)** The ranking in the prediction list for those top 3 dominant ARs observed every year. - indicates the observed variants didn’t be detected in the prediction list, NA indicates that less than 3 dominant variants with abundancy above 5% were observed that year.

Annual checking was done with results shown in Figure 1A. Firstly, the number of mutant types in each AR fluctuates over time, with a range of 5–30 variants each year. In the subsequent 5 years, the averaged strain and type coverage were seen as 96.5 and 44.8% for Ca1, 98.5 and 50.7% for Ca2, 98.7 and 17.8% for Cb, 97.6 and 56.6% for Sb, and 47.8 and 19.1% for SA (Supplementary Table 1). More importantly, the sharp contrast between low type coverage and high strain coverage suggested the ability to capture those dominant strains among offspring variants on most ARs. It was also noteworthy that the strain coverage of Sa dropped rapidly after 3 years and lost predictive performance afterward. This may be related with the long interval from the template being adopted, where Sa region might mutate at a different rate from others.

In addition, the ranking of the observed dominant mutants was also checked in the prediction list. In Figure 1B, those top circulating mutants in each AR were successfully short-listed by the prediction. Most of the time, the top 1 prevalent was forecasted as the top 1, which is particularly obvious after 2 years of template selection. For example, in 2015 and 2016, all the top 1 community-prevalent variants were predicted as exactly the top 1 excepting Sa region. Surprisingly, in 2015, all the newly emerged sub-dominant mutants (top 2) circulating in the community were successfully predicted as top 1 to top 7. In other words, the to-be-emerged mutants seemed to be foreseen 1 year before circulation. Though the model performance dropped along the time as more mutants emerged, above 5-year comparison confirmed the high possibility of forecasting future dominant mutants, at least within the subsequent 1–2 years.

## Discussion

In summary, predicting the future mutational profiles is highly desirable but challenging for rapidly mutating RNA viruses, such as influenza. Here, we compared a prediction list in 2014 with the subsequent 5-year reported variants, and the results highlighted the possibility to predict future mutants for influenza. In addition, we provided another prediction list of A/H1N1 for future seasons (Supplementary Table 2) based on the template of A/Pennsylvania/02/2021, and a prediction list of A/H3N2 (Supplementary Table 3) based on the template of A/Maryland/12239/2021, respectively. Interestingly, multiple mutations were detected involving significant property changes which might cause antigenic variations. For instance, H141Y in Ca2 (rank 7), S74F in Cb (rank 11), K163N in Sa (rank 3) for A/H1N1, or K131E in A (rank 3), F174L in D (rank 2), and so on for A/H3N2. Of note, the emergence of SARS-CoV-2 in 2020, may have dramatically affected the evolution of many respiratory viruses, including influenza virus. The model performance based on pre-SARS-CoV-2 data remains to be tested in the future. Also, current results only apply to single-site mutations in the HA ARs, while predicting the exact mutations at the strain level remains to be explored in the future. At last, though this strategy might theoretically be extended to other viruses, the feasibility needs to be re-evaluated for those newly emerged pathogens like SARS-CoV-2.

## Author contributions

ZC and TQ conceived and designed the experiments and supervised the whole project. TM constructed the model and performed the data analysis. DY collected the data. MZ helped to analyze the results. TM, TQ, and ZC wrote the manuscript. All authors reviewed and approved the manuscript.
