# Supplementary material for: Possibility of estimating future mutants for influenza: Comparison between previous prediction and subsequent years observation
Source: Front Microbiol. 2022 Oct 5;13:1031672. doi: 10.3389/fmicb.2022.1031672 (PMC9581178; doi:10.3389/fmicb.2022.1031672)
Supplement: Supplementary file 1 [file Data_Sheet_1.docx]

Supplementary Material

**Methods**

**Data resources.**

Nucleotide sequence data of influenza hemagglutinin (HA) were collected from the NCBI influenza virus resources database(Bao et al. 2008). The sequence data was filtered through three steps: 1) remove those sequences with non-standard nucleotides other than regular DNA bases of ATCG, 2) remove that incomplete HA sequences (<978 nt), and 3) Remove those sequences with reporting years other than 2015-2019. After that, a total number of 7691 HA sequences for A/H1N1 remained in our dataset. The amino acid sequences of influenza HA were translated from the above nucleotide sequence by bio-python(Cock et al. 2009), and the HA1 fragments were intercepted after multiple sequence alignment through Clustal Omega(Madeira et al. 2019).

The mutant offspring profile predicted from a template sequence with the highest abundance of 2014 for A/H1N1 was from **Supplementary Table S4** in our previous work(Xu et al. 2016).

**Brief description of the algorithm**

The “mutation-selection-ranking” strategy in our previous work(Xu et al. 2016)involves three steps:

1. Deriving the mutational transition matrix based on the dynamic evolutionary pattern from long-time accumulation of the influenza genomes and simulating the mutation profile in HA antigenic regions.

2. Simulating the potential fitness from host selection based on filtering rules, which could filter out offspring sequences that carried too many unexpected mutations.

3. Ranking the mutation profiles of the offspring sequences based on the simulated abundance after host selection.

**Evaluation parameters**

Type coverage was calculated as:

Type coverage = T_top_/T_total_ (1)

T_top_ indicates the number of observed antigenic region types correctly predicted in the simulated mutation profiles; T_total_ indicates the total number of antigenic region types actually observed every year.

Stain coverage was calculated as:

Strain coverage = S_top_/S_total_  (2)

S_top_ represents the total number of strains containing the correctly predicted antigenic region in the simulated mutation profiles; S_total_ represents the total number of HA1 strains reported every year.

**Supplementary Tables**

**Table S1. Details of the type coverage and strain coverage of A/H1N1.**

| **Period** | **AR** | **Strain Coverage** | **Type coverage** |
| --- | --- | --- | --- |
| **2015** | Ca1 | 99.1% | 50.0% |
|  | Ca2 | 98.7% | 63.6% |
|  | Cb | 99.4% | 20.0% |
|  | Sa | 99.1% | 50.0% |
|  | Sb | 98.7% | 76.9% |
| **2016** | Ca1 | 96.9% | 36.8% |
|  | Ca2 | 99.2% | 50.0% |
|  | Cb | 98.2% | 31.6% |
|  | Sa | 98.2% | 26.9% |
|  | Sb | 98.9% | 56.7% |
| **2017** | Ca1 | 88.9% | 36.4% |
|  | Ca2 | 99.0% | 64.3% |
|  | Cb | 98.8% | 18.2% |
|  | Sa | 41.8% | 13.6% |
|  | Sb | 97.2% | 48.0% |
| **2018** | Ca1 | 98.5% | 45.0% |
|  | Ca2 | 97.6% | 42.3% |
|  | Cb | 98.0% | 8.3% |
|  | Sa | 0.1% | 5.0% |
|  | Sb | 98.3% | 55.2% |
| **2019** | Ca1 | 99.4% | 55.6% |
|  | Ca2 | 98.2% | 33.3% |
|  | Cb | 99.1% | 11.1% |
|  | Sa | 0.0% | 0.0% |
|  | Sb | 95.1% | 46.2% |

**Table S2. Potential mutation profile of antigenic sites for A/H1N1 in future with A/Pennsylvania/02/2021 (the most abundant sequences in 2021) as template.**

| **CA1** | **CA2** | **CB** | **SA** | **SB** |
| --- | --- | --- | --- | --- |
| INDKGTSREPG | PHAGAKRD | LSTARS | PNKKGNSPKLNQT | TIAAQESLYQNA |
| INDKRTSREPG | PHARAKRD | LSTVRS | PNKKRNSPKLNQT | TIAAQDSLYQNA |
| INDKGTSREPR | PHAGANRD | LTTARS | PNKKGNSPNLNQT | TIAAQESLYHNA |
| INDKGTSRDPG | LHAGAKRD | LSSARS | PNNKGNSPKLNQT | TIAAQESLYQNV |
| INDNGTSREPG | SHAGAKRD | LSTASS | PNKKGNSPKLNHT | TIAVQESLYQNA |
| INDKGTSRELG | PHAGVKRD | LSPARS | PNKNGNSPKLNQT | TIVAQESLYQNA |
| INDKGTSRESG | PYAGAKRD | LRTARS | PNKKGNSLKLNQT | TIAAQKSLYQNA |
| INNKGTSREPG | PHVGAKRD | LSTAGS | LNKKGNSPKLNQT | TITAQESLYQNA |
| INDKGTSRKPG | PHAGTKRD | LPTARS | PNKKGNSSKLNQT | TIATQESLYQNA |
| IKDKGTSREPG | PHTGAKRD | LLTARS | SNKKGNSPKLNQT | TIAAQESLYQKA |
| INEKGTSREPG | PHAGAKRN | LFTARS | PKKKGNSPKLNQT | TIAPQESLYQNA |
| INDKGISREPG | PQAGAKRD | LSTAKS | PNKKGKSPKLNQT | TIPAQESLYQNA |
| INDKGTSREPA | PHAGAKRE | LNTARS | PNKKGNSPKLKQT | TIAAQQSLYQNA |
| INDKATSREPG | PHAAAKRD | LSTERS | PNKKGNSPKLNQI | TIAAQESLYRNA |
| INHKGTSREPG | PHPGAKRD | LCTARS | PNKKANSPKLNQT | TIAAQGSLYQNA |
| INDKGTSRQPG | PHAGAKRH | LITARS | PNKKGNSPKLNRT | TIASQESLYQNA |
| LNDKGTSREPG | PHAGPKRD | LGTARS | PNKKGNSPKFNQT | TISAQESLYQNA |
| INGKGTSREPG | PHAGAKRG | LYTARS | PNRKGNSPKLNQT | TIAAQETLYQNA |
| INDKGTSRGPG | PRAGAKRD | LATARS | PNKRGNSPKLNQT | SIAAQESLYQNA |
| INYKGTSREPG | PHSGAKRD | LWTARS | PNKKGNSPRLNQT | TIAAQASLYQNA |
| INDRGTSREPG | PHAGARRD | LSSVRS | PNKKVNSPKLNQT | TIAAQESLYQTA |
| INDKGTSREPV | PHAGSKRD | LTTVRS | PNKKGNSPKLSQT | TTAAQESLYQNA |
| ISDKGTSREPG | PHAVAKRD | LSTVSS | PNKKGSSPKLNQT | TIAAQVSLYQNA |
| VNDKGTSREPG | PHAGAKRY | LSPVRS | PSKKGNSPKLNQT | TIAAQESIYQNA |
| INDKVTSREPG | PHAGAERD | LRTVRS | PNKKGNSPKLNQA | TMAAQESLYQNA |
| INDKGASREPG | PNAGAKRD | LSTVGS | PDKKGNSPKLNQT | TIGAQESLYQNA |
| IDDKGTSREPG | PHAGAKSD | LTSARS | PNKEGNSPKLNQT | TIAAQESLYQNG |
| INDKGTTREPG | THAGAKRD | LTTASS | PNKKGNSPELNQT | TIAGQESLYQNA |
| INDEGTSREPG | PHAGATRD | LSSASS | PNKKGDSPKLNQT | TIADQESLYQNA |
| INDKGSSREPG | PHAGAKRA | LLTVRS | PNEKGNSPKLNQT | TIAAQESLYQND |
| INDKGTSRETG | PPAGAKRD | LPTVRS | PNKKGNSPKLDQT | NIAAQESLYQNA |
| INDKGTSSEPG | PHAGAQRD | LKTARS | PNKKGNTPKLNQT | TIDAQESLYQNA |
| INDTGTSREPG | PHAGAKPD | LTPARS | PNKKGNSPKLNQS | TIAAQENLYQNA |
| INDKGTSRAPG | PHASAKRD | LFTVRS | PNKKGNSPKLNKT | KIAAQESLYQNA |
| INAKGTSREPG | PLAGAKRD | LLTTRS | TNKKGNSPKLNQT | TIAAQESLYQNE |
| ITDKGTSREPG | PHAGAKRV | LSPASS | PNKKGNSTKLNQT | TIAEQESLYQNA |
| IHDKGTSREPG | PHADAKRD | LFTTRS | PNKKGNSPTLNQT | TIEAQESLYQNA |
| INDKGPSREPG | PHAGAKLD | LVTARS | PNKTGNSPKLNQT | TIAAQEGLYQNA |
| INDQGTSREPG | PHAEAKRD | LSTVKS | PNTKGNSPKLNQT | TIAAQEALYQNA |
| TNDKGTSREPG | PHAGAKGD | LNTVRS | PNKKGNSPKLNPT | TSAAQESLYQNA |
| INDKGTSPEPG | PHAGAKCD | LSTAES | PNKKGNSPKLTQT | TIAAQESRYQNA |
| INDKSTSREPG | PHACAKRD | LSTNRS | PNKKGTSPKLNQT | TIAAQDSLYHNA |
| INDKGTSRVPG | RHAGAKRD | LTTAGS | PTKKGNSPKLNQT | TIAVQESLYHNA |
| INDKGTSREPS | AHAGAKRD | LRTASS | PNKQGNSPKLNQT | TIAVQDSLYQNA |
| INVKGTSREPG | PHAGGKRD | LSTKRS | PNKKGNSPKLNQP | TIAAQDSLYQNV |
| IIDKGTSREPG | PDAGAKRD | LRSARS | PNKKGNSPQLNQT | TIVAQDSLYQNA |
| INDKGTSLEPG | PHGGAKRD | LLIARS | PNQKGNSPKLNQT | TIVAQESLYHNA |
| IYDKGTSREPG | PHAGAKHD | LHTARS | PHKKGNSPKLNQT | TIAAQESLYHNV |
| INDKGTSREPD | PHAGDKRD | LSSAGS | PNKKGNSPKLHQT | TIAVHESLYQNA |
| INDKDTSREPG | PHAGAKQD | LFIARS | PNKKGHSPKLNQT | TIAAQRSLYQNA |

**Table S3. Potential mutation profile of antigenic sites for A/H3N2 in future with A/Maryland/12239/2021 (the most abundant sequences in 2021) as template.**

| **A** | **B** | **C** | **D** | **E** |
| --- | --- | --- | --- | --- |
| NSKNSSSSS | TSNIQDKSAQ | EGSRK | EFRKVIIL | GQKEIN |
| NSRNSSSSS | TSNIQDKSTQ | KGSRK | ELRKVIIL | GQKKIN |
| NSENSSSSS | TSNIQNKSAQ | GGSRK | KFRKVIIL | GRKEIN |
| NSKSSSSSS | TSNIQDKSVQ | EGSRR | EFRKIIIL | GQKEIS |
| SSKNSSSSS | TSNIQDKSAR | EGSRE | GFRKVIIL | GQEEIN |
| NSKDSSSSS | TSNIQGKSAQ | ERSRK | EFRKVVIL | EQKEIN |
| DSKNSSSSS | TSNVQDKSAQ | EESRK | EFRKVIVL | GHKEIN |
| NSKKSSSSS | TSNIQDRSAQ | ESSRK | EFRRVIIL | GKKEIN |
| KSKNSSSSS | TSDIQDKSAQ | EDSRK | EFREVIIL | GQKELN |
| NSNNSSSSS | TSNTQDKSAQ | EGSRN | ESRKVIIL | GQKEMN |
| NSKNPSSSS | TSNIQEKSAQ | DGSRK | EFRKVTIL | GLKEIN |
| NSKNSPSSS | TSKIQDKSAQ | EGPRK | EFRKVITL | GQKEII |
| NSKNSSSSP | TSNIQDKSAH | EGSQK | EFRKAIIL | GQKEIT |
| NSKNSSSPS | TSNIQDNSAQ | EGSKK | DFRKVIIL | GPKEIN |
| NSKNSSPSS | TPNIQDKSAQ | EGSHK | EFRKVIIF | GEKEIN |
| NPKNSSSSS | TNNIQDKSAQ | EGNRK | EFRNVIIL | GRKKIN |
| NNKNSSSSS | TSNIQDKNAQ | EGLRK | EFRKVIIP | GQKKIS |
| NSKNSNSSS | TLNIQDKSAQ | EVSRK | EIRKVIIL | GQEKIN |
| NSKNSSSNS | TFNIQDKSAQ | EGFRK | EYRKVIIL | EQKKIN |
| NSKNSSNSS | TSNLQDKSAQ | EGSCK | EFQKVIIL | NQKEIN |
| NSKNSSSSN | TTNIQDKSAQ | EGTRK | EFKKVIIL | KQKEIN |
| NSKNNSSSS | TSNILDKSAQ | EGSGK | EFHKVIIL | GRKEVN |
| NLKNSSSSS | TSNMQDKSAQ | EGSSK | EFRKVILL | GRKGIN |
| NSKNSSLSS | TSNNQDKSAQ | VGSRK | EFRKVLIL | GRREIN |
| NSKNLSSSS | TGNIQDKSAQ | EGSLK | EFCKVIIL | GREEIN |
| NSKNSLSSS | TRNIQDKSAQ | EGGRK | EFGKVIIL | GRKEID |
| NSKNSSSLS | PSNIQDKSAQ | EGRRK | EFRKLIIL | GRKEIS |
| NSKNSSSSL | TSNIQDQSAQ | AGSRK | EFSKVIIL | GQGEIN |
| NSKNSSSFS | TSNFQDKSAQ | EGSRT | EFRKVMIL | GQKEIR |
| NSKNSFSSS | TYNIQDKSAQ | EGSRQ | EFRKVIML | GQEEIS |
| NSKNFSSSS | TSNKQDKSAQ | EGSWK | EFRKVIII | RRKEIN |
| NFKNSSSSS | TINIQDKSAQ | ECSRK | VFRKVIIL | GHKKIN |
| NSKNSSSSF | TCNIQDKSAQ | EGYRK | EFRKMIIL | GRKETN |
| NSKNSSFSS | TSNSQDKSAQ | EGSRI | EFRKVINL | GKKKIN |
| NSKNSSSTS | TANIQDKSAQ | EGIRK | EFRKVNIL | GYKEIN |
| NSKNSSTSS | TSNIQNKSTQ | EGSRM | EFLKVIIL | SRKEIN |
| NSKNSSSST | TSNIQNKSVQ | QGSRK | EFRKVIIS | ERKEIN |
| NSKNTSSSS | TSNIQDKSTR | EASRK | AFRKVIIL | DRKEIN |
| NSKNSTSSS | TSNIQNKSAR | EGCRK | EFRTVIIL | EQKEIS |
| NTKNSSSSS | TSNVQDKSTQ | EGARK | EFRQVIIL | EQEEIN |
| ISKNSSSSS | TSNIQGKSTQ | EWSRK | EFRKEIIL | GRKEIK |
| NSKISSSSS | TSNIQNRSAQ | EGSPK | EFRKDIIL | GQKKLN |
| NSKYSSSSS | TSNVQNKSAQ | EGSIK | EFWKVIIL | GRKDIN |
| YSKNSSSSS | TSNIQDRSTQ | RGSRK | EFRKVIFL | GRNEIN |
| NSKNSSSGS | TSNIQDKSIQ | EGSMK | EFRKVFIL | GWKEIN |
| NSKNGSSSS | TSDIQDKSTQ | KGSRR | EFRKFIIL | GHREIN |
| NSKNSSGSS | TSDIQNKSAQ | KGSRE | EFRIVIIL | GHKGIN |
| NSKNSGSSS | TSNIQDKSVR | KRSRK | EFRKVKIL | GHKEIS |
| NSKNSSSSG | TSNTQDKSTQ | EGSTK | EFRKVIKL | GHKEID |
| NRKNSSSSS | TSNIQGKSVQ | EKSRK | EFRKVIIQ | GHKEVN |

**Reference**

Bao, Y., P. Bolotov, D. Dernovoy, B. Kiryutin, L. Zaslavsky, T. Tatusova, J. Ostell, and D. Lipman. 2008. 'The influenza virus resource at the National Center for Biotechnology Information', *J Virol*, 82: 596-601.

Cock, P. J., T. Antao, J. T. Chang, B. A. Chapman, C. J. Cox, A. Dalke, I. Friedberg, T. Hamelryck, F. Kauff, B. Wilczynski, and M. J. de Hoon. 2009. 'Biopython: freely available Python tools for computational molecular biology and bioinformatics', *Bioinformatics*, 25: 1422-3.

Madeira, F., Y. M. Park, J. Lee, N. Buso, T. Gur, N. Madhusoodanan, P. Basutkar, A. R. N. Tivey, S. C. Potter, R. D. Finn, and R. Lopez. 2019. 'The EMBL-EBI search and sequence analysis tools APIs in 2019', *Nucleic Acids Res*, 47: W636-W41.

Xu, H., Y. Yang, S. Wang, R. Zhu, T. Qiu, J. Qiu, Q. Zhang, L. Jin, Y. He, K. Tang, and Z. Cao. 2016. 'Predicting the Mutating Distribution at Antigenic Sites of the Influenza Virus', *Sci Rep*, 6.
